# A multicentre survey of knowledge and implementation of radiation protection techniques in cardiac cath-lab medical personnel

**DOI:** 10.1186/s43044-024-00492-4

**Published:** 2024-06-03

**Authors:** Amr Mansour, Noha M. Gamal, Ali Tohamy, Adham Abdeltawab

**Affiliations:** 1 Cardiology Department, Ain Shams University Hospital, 62 Hegaz St, Abbassia, Heliopolis, Cairo, Egypt; 2 Cardiology Department, Assiut University, Asyut, Egypt

**Keywords:** Radiation awareness, Radiation hazards, Radiation protection, Cardiology Cath-Lab, Survey

## Abstract

**Background:**

Awareness of radiation hazards and methods to reduce radiation dose is a sine qua non for all staff working in the cath-lab for their own safety and their patient’s safety.

**Results:**

There were large variations in the implementation of radiation protection techniques with overall inadequate radiation risk knowledge. Some members of the cath-lab team are at higher risk of radiation-induced side effects, including the fellows, nurses, technicians, and anaesthesiologists because they spent longer time in the cath-lab and/or their position in relation to the source of radiation. About 10% of the participants have reported different health problems potentially induced by radiation exposure.

**Conclusions:**

There is lack of radiation risks knowledge with inadequate radiation protection practice among cath-lab team. Some members such as fellows, nurse, technicians, and cardiac anaesthesiologist are at higher risks. They represent the forgotten members of the Cath-Lab team.

**Supplementary Information:**

The online version contains supplementary material available at 10.1186/s43044-024-00492-4.

## Background

The increase in use of minimally invasive procedures in the past few decades caused in an exponential growth in fluoroscopy-guided catheter-based cardiology procedures. Staff members of cardiac catheterization laboratories are at higher risk of multiple occupational hazards, among which are the risks of radiation exposure [1]. With more dependence on catheter-based procedures, the adverse effects of radiation exposure to the patient, operator, and ancillary staff have been a subject of concern [1]. There is no minimum safety threshold for radiation and its adverse effects occur in a linear, dose-dependent risk [2]. Radiation injury risk is much higher in interventional cardiologists as compared to clinical cardiologists given their chronic radiation exposure in cardiac catheterization laboratories. They develop somatic DNA damage and chromosomal abnormalities at a higher frequency [3]. They also develop significantly higher incidence of brain tumours and cataract [4, 5]. Similarly, anaesthesiologists working in the cardiac catheterization laboratory are exposed to significantly more radiation compared to those working in operation rooms [6]. A personal dose metre placed on an anaesthesia machine has been shown to receive 15 times the radiation compared to the dosimeter worn by a scrub nurse [7].

Many protection shields and techniques can be adopted to reduce radiation exposure and proper technique and positioning in cath-labs, together with proper protection and shielding can significantly reduce risk to personnel working in cath-lab [8, 9].

Hence, adequate knowledge of the radiation hazards and how to reduce them is a sine qua non for all the medical staff team members to minimize the potential risks to the patients and medical personnel. This is also needed to apply the risk–benefit assessment and to reinforce the principles of justification and optimization in clinical practice [9].

Thus, our aim was to evaluate the cardiac catheterization laboratories staff knowledge of radiation exposure risks to themselves and to the patients and to assess their daily implementation of radiation protection techniques.

## Methods

A dedicated survey was designed for this study. This was done through a questionnaire. The survey questions were reviewed by 3 senior expert interventional cardiologists before approving their final version.

The questions were designed for assessing the staff’s knowledge of the radiation hazards and potential risks to the operators and the patients and documenting the real-life implementation of radiation protection techniques of the participants (questionnaire attached as amendment 1).

We targeted all the cardiac catheterization laboratory team members including the physicians, nurses, and the technicians.

The survey was available in two identical formats: a printed paper format and an online electronic version to encourage the participation of the targeted population according to their own preference. The questions were written in both English and Arabic languages to ensure that all the participants understand the meaning and exact purpose of each question. The medical terms were translated using the unified medical dictionary (UMD) version approved by the World Health Organization (WHO).

The survey was sent through the emails to the participants; it was also available on multiple social media platforms and as an online link in the Egyptian cardiologists’ groups, and private institutional cardiologists’ groups. Two electronic reminders were sent in each site. The printed version was distributed and filled during the Egyptian society of cardiology largest annual conference, Cardio Egypt conference.

A custom-made sheet was made to include the participants’ demographic data, affiliation, the number of days per week spent in the cath-lab, and the average number of procedures performed per week. We also included the main subspecialty for the physicians, and the work nature for nurses and technicians.

After collection, revision, coding of the data, it was analysed using the Statistical Package for Social Science (IBM SPSS) version 23.

## Results

From a total number of 860 surveys sent and distributed, we received 492 responses, representing 57.2%.

Staff members working in more than 20 different cardiology cath-labs from almost all Egyptian governorates had participated in this study. In total, 398 participants (80.9%) were males, and 94 participants (19.1%) were females.

**Fig. 1:**
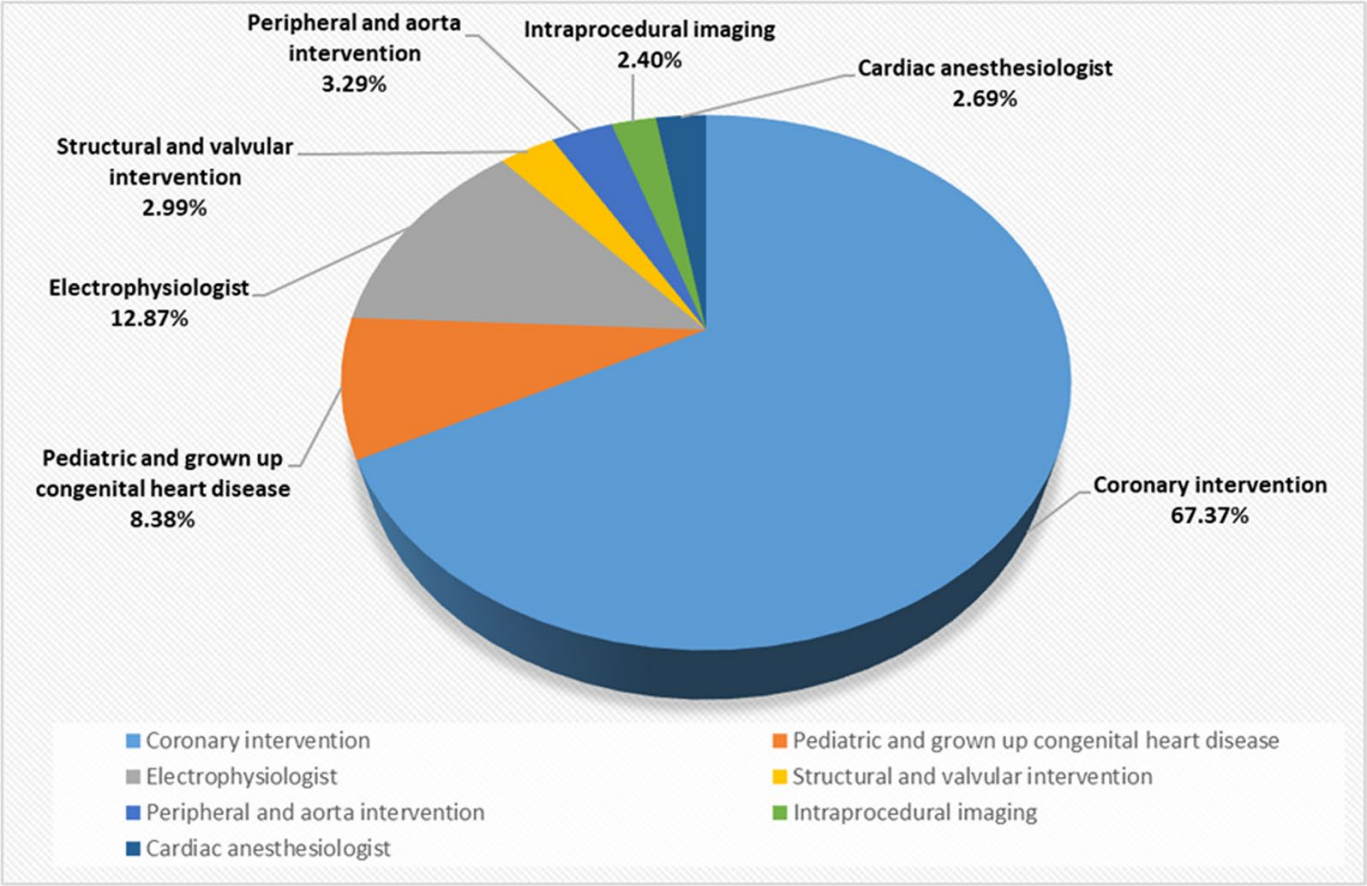
The subspecialties of the physicians participating in the study

All medical staff members working in the cath-lab including technicians, scrub, and circulating nurses-had actively participated in this study (Table 1). Interventional cardiologists from all subspecialties, interventional cardiology imaging staff, and cardiac anaesthesiologists were also included in the survey (Fig. 1).

**Table 1:** Comparing of the number of days and cases performed per week between nurses, technicians and physicians

	Physicians	Nurses	Technicians	Test value	*P*-value
	No. = 334	No. = 73	No. = 85		
*What are the average days per week do you work in the Cath. Lab.?*					
Mean ± SD	2.57 ± 1.05	4.27 ± 1.43	4.95 ± 0.67	211.803•	< 0.01
Range	1–6	2–7	3–7		
*How many procedures per week (on average) do you do in the Cath. Lab.?*					
Median	7 (5–10)	20 (15–25)	29 (23–33)	176.811‡	< 0.01
(IQR) Range	1–60	4–70	8–170		

Standard deviation (SD), interquartile range (IQR)

*P*-value > 0.05: nonsignificant (NS); *P*-value < 0.05: significant (S); *P*-value < 0.01: highly significant (HS)

•One-way ANOVA test; ‡: Kruskal–Wallis test

Among the cardiologists participated in the survey there were 97 cardiology consultants (29.04%), 126 fellows (37.72%), and 111 residents (33.23%).

The mean number of working days in the cath-lab among the whole studied population per week was 3.24 ± 1.45 days, while the mean number of procedures attended was 10 ± 14 cases per week.

We found that nurses and technicians significantly work a greater number of days and attend a greater number of procedures per week when compared to the whole physicians’ group with *P* value < 0.01 (Table 1); however, there was no significant difference between them and the residents and fellows with *P*-value 0.21.

**Fig. 2:**
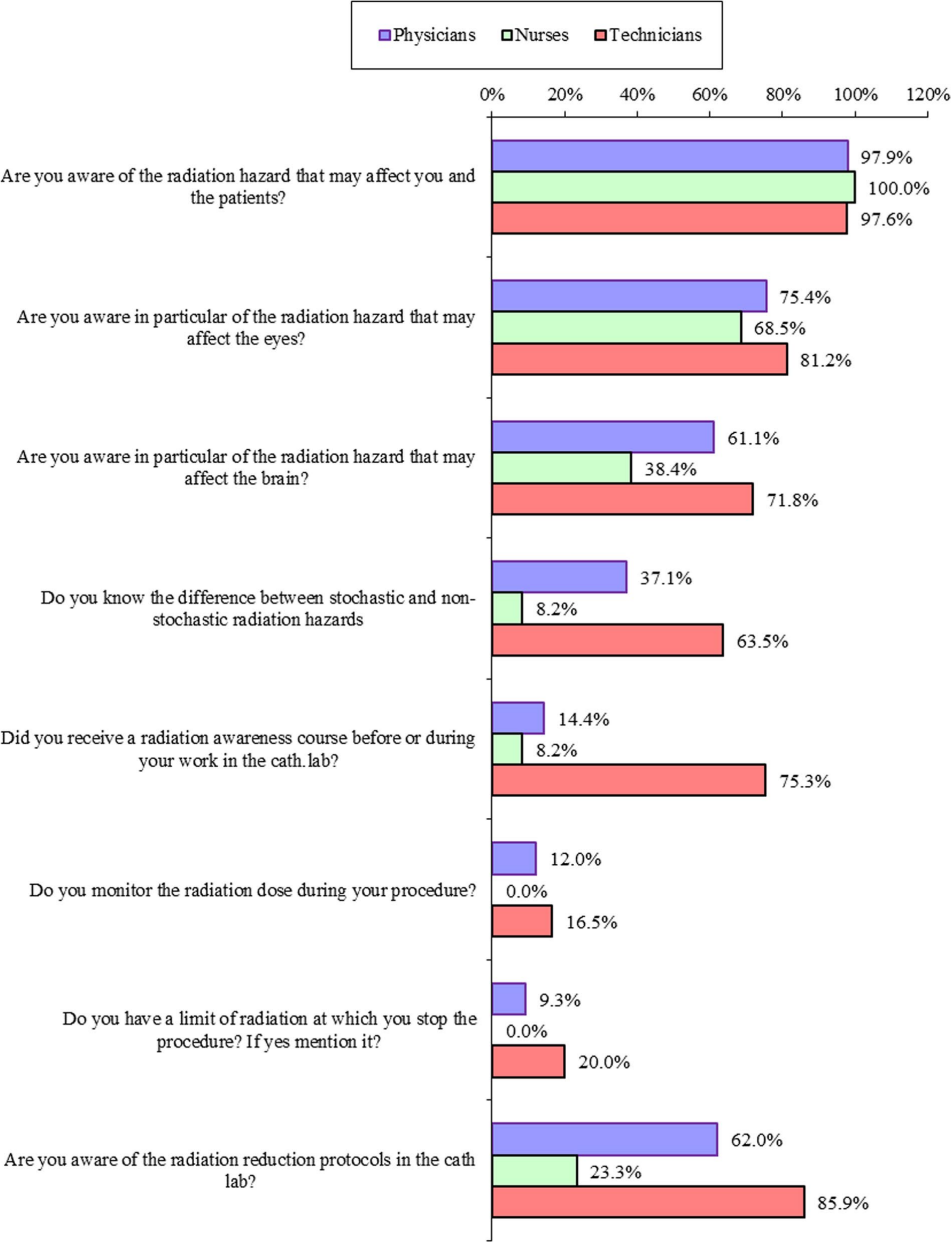
Assessment of the awareness of the participants to the risks of radiation to the operators and the patients showing the percentage of personnel who answered yes to the question in the study groups

We found that the technicians were more knowledgeable about the theoretical risks of radiation when compared to the physicians and nurses with *P* value < 0.01. (Fig. 2).

**Fig. 3:**
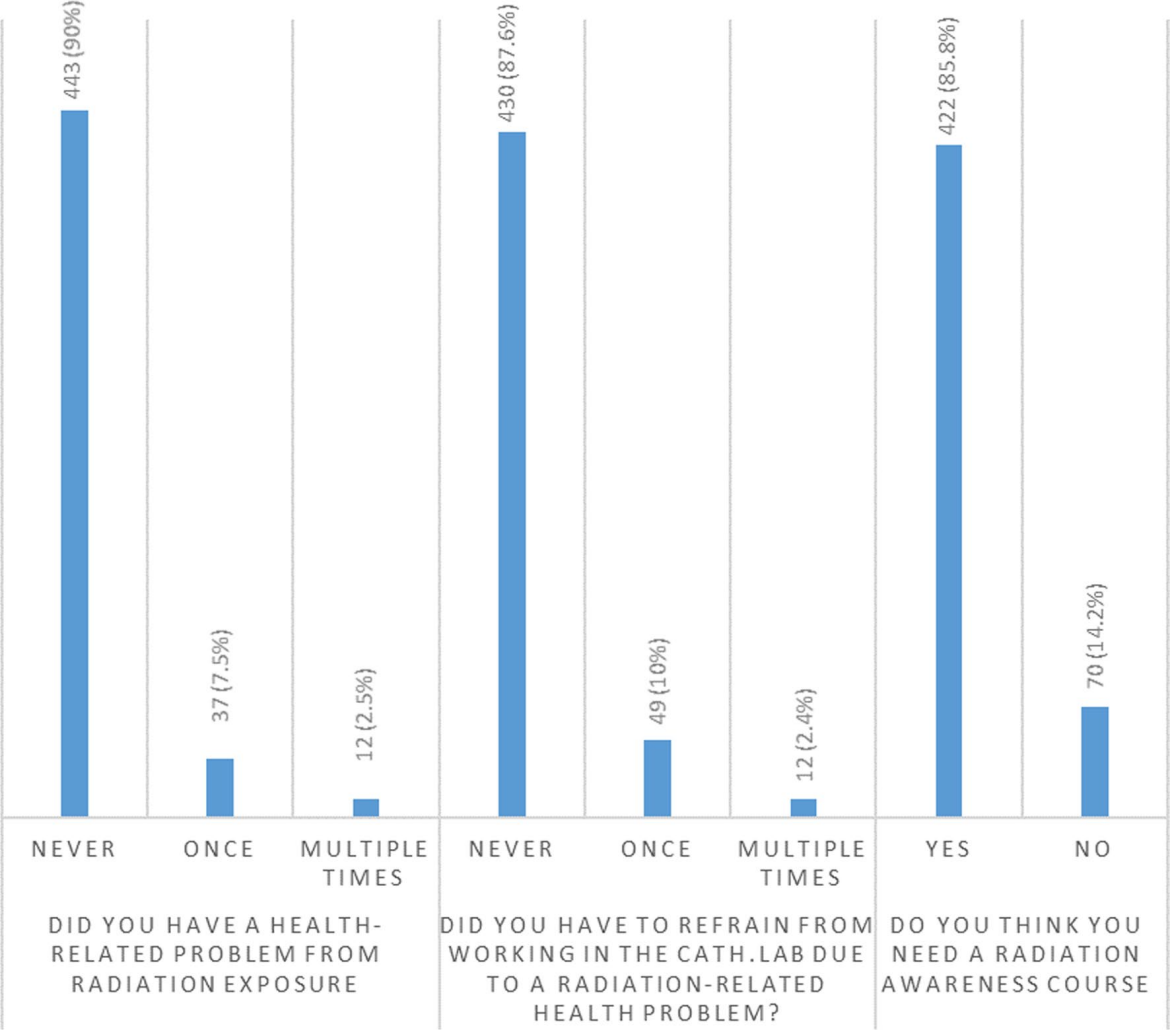
Incidence of radiation-induced health problems, abstinence from working in Cath. Lab., and the need for radiation course among the study population

Only 9.3% of the physicians and 20% of the technicians mentioned a maximum allowable limit of radiation dose per case after which they stop the procedure unless clinically impossible according to the patient’s condition, Fig. 3. However, 92% of these physicians and 95% of the technicians failed to specify a clear cut-off number to this limit, 2% of the physicians mentioned a procedural time rather than radiation dose as their limit, 1.5% mentioned that the cut-off limit is 7 Gy, 3% mentioned that it ranges from 8 to 10 Gy, and 1.5% mentioned 4–5 Gy.

62.2% of the physicians, 23.3% of the nurses, and 85.9% of the technicians were aware of the radiation reduction protocols in the cath-lab such as table height, use of Fluro store, collimation, and modification of magnification, etc.; however; we found significant heterogenicity among the study groups in the regular implementation of these techniques with general inadequate safe practice among the whole study population (Table 2) with only 9.6% of the physicians, 11.8% of the technicians implement the radiation reduction techniques regularly during work, 2.1% of the physicians, 31.5% of the nurses, and 44.7% of the technicians wear the dosimeter regularly, and only 15% of them follow up the dosimeter analysis data.

**Table 2:** Assessment of the implementation of radiation protection precautions in the Cath. Lab

	Doctors		Nurses		Technicians		Test value*	*P*-value
	No	%	No	%	No	%		
*How often do you wear protective aprons all the time in the cath-lab?*								
Never	2	.6%	10	13.7%	10	11.8%	234.859	< 0.01
Rarely	3	.9%	21	28.8%	0	0.0%		
When I remember	0	0.0%	10	13.7%	4	4.7%		
Most of the time	50	15.0%	16	21.9%	32	37.6%		
Always	279	83.5%	16	21.9%	39	45.9%		
*How often do you wear the protective goggles?*								
Never	212	63.5%	50	68.5%	57	67.1%	34.166	< 0.01
Rarely	64	19.2%	17	23.3%	9	10.6%		
When I remember	33	9.9%	6	8.2%	3	3.5%		
Most of the time	17	5.1%	0	0.0%	5	5.9%		
Always	8	2.4%	0	0.0%	11	12.9%		
*How often do you wear the thyroid protector (neck collar)?*								
Never	96	28.7%	32	43.8%	16	18.8%	25.330	< 0.01
Rarely	88	26.3%	12	16.4%	19	22.4%		
When I remember	64	19.2%	6	8.2%	17	20.0%		
Most of the time	42	12.6%	11	15.1%	23	27.1%		
Always	44	13.2%	12	16.4%	10	11.8%		
*How often do you wear the overhead protective aprons?*								
Never	281	84.1%	73	100.0%	81	95.3%	21.622	< 0.01
Rarely	34	10.2%	0	0.0%	3	3.5%		
When I remember	7	2.1%	0	0.0%	0	0.0%		
Most of the time	10	3.0%	0	0.0%	0	0.0%		
Always	2	.6%	0	0.0%	1	1.2%		
*How often do you use the table’s lower radiation shield (curtain) in the cath-lab?*								
Never	97	29.0%	16	21.9%	21	24.7%	36.105	< 0.01
Rarely	101	30.2%	33	45.2%	15	17.6%		
When I remember	41	12.3%	6	8.2%	6	7.1%		
Most of the time	73	21.9%	6	8.2%	30	35.3%		
Always	22	6.6%	12	16.4%	13	15.3%		
*How often do you use the table’s upper protection glass shield in the cath-lab?*								
Never	107	32.0%	47	64.4%	23	27.1%	80.780	< 0.01
Rarely	108	32.3%	17	23.3%	14	16.5%		
When I remember	55	16.5%	6	8.2%	4	4.7%		
Most of the time	46	13.8%	2	2.7%	34	40.0%		
Always	18	5.4%	1	1.4%	10	11.8%		

37% of the physicians have mentioned that they had worked at least once before in the cath-lab without wearing the protective apron. Emergency situations such as pericardial tamponade, complicated procedures like ST-segment elevation myocardial infarction, balloon mitral valvuloplasty, and severe symptomatic patients with bradycardia during transient wire implantation requiring resuscitation, intubation, or offering emergency help to colleagues were the most common causes mentioned by the physicians, followed by temporarily orthopaedic problems including back pain, neck pain due to the apron’s heavy weight.

43% of the nurses and technicians reported also working occasionally without aprons, with orthopaedic problems resulted from wearing the aprons for a long time being the commonest cause.

We surprisingly found that 3.5% of the nurses and 4.8% of the technicians do not wear the protective apron during the working in the cath-lab lately because they were unable to bear the heavyweight of the aprons due to chronic back and neck orthopaedic problems, or previous orthopaedic surgeries in the vertebral column.

This attitude was fuelled by a sensation of relative security because of their position at the end of the table relatively away from the source of radiation.

Seven participants (1.4%) mentioned that they have a patient who had experienced a radiation-induced complication, namely radiation-induced skin burn and ulcer. In our study, 10% of the participants reported to have health problems that are potentially induced by radiation exposure and 2.5% of them had these health problems multiple times, Figure 3.

Among the most mentioned health problems were recurrent sensation of fatigue and body aches (45%), neutropenia (18%), recurrent unexplained oral ulcers (12%), recurrent upper respiratory tract, and chest infection (6%). One case had experienced reactivation of herpes zoster infection, one case reported thrombocytopenia, one case had a radiation-induced oedema, and one case reported to have a haematological malignancy—although not proven to be related directly to radiation exposure.

12.4% of the participants had to refrain from working in the cath-lab temporarily due to these health-related problems, Figure 3

At the end of the survey, most of the participants—85%—thought that they need radiation awareness course.

## Discussion

This survey represents the real-life’s current knowledge of the radiation hazards and describes the daily radiation protection practice implementation of the cardiac cath-lab staff members in Egypt.

Unlike most of the previously conducted similar studies which focused on the interventional cardiologists and cardiac fellows, we intended to involve all cath-lab staff members—including the scrub and circulating nurses, and technicians—because they are an integral part of cath-lab team, and they are usually underrepresented in most of the studies. We also included cardiac anaesthesiologists, interventional cardiology imaging staff because they recently became a fundamental part of the team working in the cath-lab during a lot of complex congenital paediatric, structural, electrophysiological, and adult interventional procedures.

The fact that only minority of the participants uses dosimeter and even less percentage of them calculates their radiation exposure dosimeter results regularly did not allow us to accurately calculate the amount of radiation to which our survey cohort expose. Nurses and technicians scrub in a higher number of cases and spent more days working in the cath-lab which exposes them to a higher dose of radiation when compared to the whole group of physicians. We also found that among the group of physicians, residents and fellows were the highest subgroups in terms of exposure to radiation because they were exposed to almost the same workload as nurses and technicians when compared to the consultants with *P*-value < 0.01.

The same results were reported by the study conducted by Vlastra W et al. [10] in 2019 who concluded that fellows-in-training were exposed to a 34% higher relative radiation exposure compared with the staff interventional cardiologists (*p* = 0.025). This signifies that these subgroups are at relatively higher risk of radiation hazards, requiring a special attention to both awareness and protection.

Our results revealed that in most of the institutions it is neither obligatory nor a prerequisite for the physicians and nurses to take a formal course addressing radiation hazards before joining the work in the cath-lab, therefore a considerable percentage of them lacks a clear theoretical background about the radiation risks, this was concluded from their deficient information of the radiation’s hazards on the eye, brain, and lack of adequate knowledge to differentiate between nonstochastic and stochastic radiation effects.

On the contrary, the technician’s theoretical knowledge of the radiation risks was better than that of the physicians and nurses (*p*-value < 0.01) as evidenced by their answers of this part of the survey. This is mostly due to their academic background studies before working in the cath-lab that gives them a lot of information about radiation’s hazards.

We also found that regular implementation of radiation reduction techniques during working in the cath-lab such as the use of Fluro store, collimation, etc., was only performed by a minority of the participants in the study, 9.6% of the physicians and 11.8% of the technicians, with significant variability between the study groups and within the same group, with overall insufficient use of radiation reduction techniques. This is because of a combination of lack of information and participants attitude regarding radiation hazards.

This concurs with results reported by multiple previous studies in the cardiology field and also in the interventional radiology field including a multicontinental survey [11], national surveys from South Africa [12, 13], Thailand [14], Turkey [15], Italy [16] and Lithuania [17], and USA [18]. All have consistently reported that there is a lack of awareness of the radiation risks, variation in the implementation of the precautionary maneuverers during working in the cardiac cath-labs, and variation in using protective devices regularly during work.

These cumulative data clearly indicate that the lack of radiation awareness, and inadequate implementation of protection techniques and regular use of protection devices from radiation such as the ceiling-suspended leaded shield and leaded eyeglasses, is a global problem in both the developed and developing countries.

On the contrary to the previously mentioned studies, we did not include only cardiologists, but we also included nurses, technicians, interventional cardiac imagers, and cardiac anaesthesiologists in our survey because they represent a fundamental member of the cath-lab team in the current era of complex structural, paediatric, electrophysiological procedures.

Nurses and technicians work more days and actively participates in a larger number of cases than consultant cardiologists, and they are underrepresented in most of the studies. We would like also to mention that cardiac anaesthesiologists are at a higher risk of radiation exposure because of their relatively fixed position near the patient’s head during the procedures just adjacent to the source of radiation and image intensifier, and their inability to maintain a safe distance from the radiation source due to space limitation, and lack of dedicated table shield in most of the circumstances. These remarks were highlighted previously by Biso et al. [19] who found that when we put a personal dosimeter on an anaesthesia machine, it receives 15 times more radiation compared to a dosimeter worn by a scrub nurse. This makes cardiac anaesthesiologists a hidden high-risk group for radiations-induced complications [19].

One of the commonest occupational health risks among cardiac cath-lab workers is the orthopaedic illnesses [20]. They represent a real-life problem that needs special attention. Orthopaedic illnesses were mentioned as a common cause of working temporarily without apron among the physicians, and they were the commonest cause for working without apron among the nurses and technicians.

The attitude of inadequate implementation of radiation protection techniques by technicians and nurses was supported by their false sensation of relative safety due to their relatively distant position away from the radiation source at the end of the table. However, we would like to mention that the higher number of days they work in the cath-lab per week and the higher number of cases they attend per week were not taken into their account.

## Conclusions

There is a generalized lack of knowledge regarding the radiation’s hazards among the cath-lab medical staff.

We found also inadequate implementation of radiation reduction techniques, with inadequate monitoring of the radiation doses and use of radiation protective devices.

Cardiology fellows, cardiac catheter scrub and circulating nurses, technicians and cardiac anaesthesiologists are apparently at higher risk of radiation-induced health problems because of higher radiation exposure when compared to the consultants, they are underrepresented in most of the previously conducted surveys and studies. We believe that they require special attention and dedicated courses.

Reduction of radiation exposure hazards is needed by using new radiation protection devices that have minimal or no orthopaedic-induced health problems to the medical staff. Also, robotic interventions have recently become a part of daily practice in multiple centres reducing the medical staff radiation exposure; this will expand more in the future.

### Recommendation

We recommend more strict regulations in different cath-labs as regards staff education and making radiation safety awareness an integral part of the licensing procedure for all working personnel in cath-lab. Also, proper shielding should be available in all cath-labs with minimum tolerance to lack of proper aprons and shielding equipment with frequent monitoring of cath-labs as regards availability and awareness of staff as regards radiation hazards and methods of their reduction. Promotion of new tools as robotic interventions might also reduce radiation exposure and is hazards.

### Study limitations

Main limitation as regards current study is small number of participants and smaller representation of cath-labs in the private sector.

### Supplementary Information

Additional file 1. Attachment.

## Data Availability

The datasets used and/or analysed during the current study are available from the corresponding author on reasonable request.

## Abbreviations

Gy: Gray
SD: Standard deviation
IQR: Interquartile range
