# Supplementary material for: A multicentre survey of knowledge and implementation of radiation protection techniques in cardiac cath-lab medical personnel
Source: Egypt Heart J. 2024 Jun 3;76:69. doi: 10.1186/s43044-024-00492-4 (PMC11147976; doi:10.1186/s43044-024-00492-4)
Supplement: Supplementary file 1 — Additional file 1. Attachment. [file 43044_2024_492_MOESM1_ESM.pdf]

Radiation awareness survey

a survey to assess awareness of radiation hazards awareness among cath lab workers

Name/ full name or initials *الاسم أ و الاحرف الاولي فقط

Gender *

- Male
- Female

country of work *بلد العمل

Place of Work (affiliation) *مكان العمل

Email Address:

What is your job in the cath.lab? *

- Physician
- Nurse
- technician

the main subspeciality (for physician only)) سؤال للأطباء(التخصص

- Adult coronary intervention
- Pediatric intervention
- EPS
- structural intervention
- intraprocedural imaging (IVUS, OCT, TEE)
- other (mention in the description)

other (mention)

What are the average days per week do you work in the Cath. Lab.? *متوسط عدد الايام في الاسبوع

How many procedures per week (on average) do you do in the Cath. Lab.? متوسط عدد الحالات في الأسبوع*

are you aware of the radiation hazard that may affect you and the patients? هل انت علي درايه بمخاطر الاشعاع عليك و علي المرضي*

- yes
- no

are you aware in particular of the radiation hazard that may affect the eyes? هل انت علي درايه بمخاطر الاشعاع علي العين*

- yes
- no

are you aware in particular of the radiation hazard that may affect the brain? هل انت علي درايه بمخاطر الاشعاع علي المخ*

- Yes
- No

do you know the difference between stochastic and non-stochastic radiation hazards هل تعرف الفرق بين مخاطر الاشعاع العشوائية و الغير عشوائيه ***?**

- Yes
- No

7. Did you receive a radiation awareness course before or during your work in the cath. lab? *هل حضرت ورشه عمل للتوعيه بمخاطر الاشعاع

- Yes
- No

Do you monitor the radiation dose during your procedure? هل تتابع كميه الاشعاع المنبعثه من جهاز القسطرة اثناء العمل*
- Yes
- No
- sometimes

Do you have a limit of radiation at which you stop the procedure? If yes mention it? هل يوجد حد اقصي من الاشعاع الذي تتوقف بعده عن العمل في الحاله*
- yes
- No

if you answer yes to the previous question, what is this limit? ما هو الحد الأقصى من الاشعاع الذي تتوقف عنده

Are you aware of the radiation reduction protocols in the Cath lab? ه ل تعلم كيفية تقليل الاشعه المنبعثه من جهاز القسطره*
- Yes
- No

How often do you implement the radiation reduction protocols in the Cath lab? هل تفعل اجراءات تقليل الاشعه المنبعثه من جهاز القسطره*
- always
- most of the time
- when i remember
- rarely
- Never

Do you put on a dose calculating device in the Cath lab? dosemeter, film badge هل ترتدي جهاز قياس مستوي الاشعاع*
- always
- most of the time
- when I remember
- rarely
- never

Do you monitor your weekly or monthly radiation doses? * ه ل تحلل نتائج جهاز قياس مستوي الاشعاع
- always
- most of the time
- when I remember
- rarely
- never

Did you reach the upper limit of your weekly/monthly radiation before? هل وصلت للحد الأقصى من كميه الاشعاع المسموح التعرض لها من قبل*
- multiple times
- once
- do not know
- never

How often do you wear protective aprons all the time in the cath lab? هل ترتدي الابرون في غرفه القسطرة*
- always
- most of the time
- when I remember
- rarely
- never

Mention the cause that made you not able to wear the apron ان وجد (اذكر اسباب عدم ارتداء الابرون

How often do you wear the protective goggles? *هل ترتدي النظارات الواقيه

- ○ always
- ○ most of the time
- ○ when I remember
- ○ rarely
- ○ never

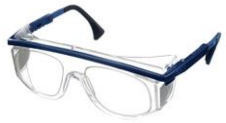

How often do you wear the thyroid protector (neck collar)? *هل ترتدي واقي الرقبه

- ○ always
- ○ most of the time
- ○ when I remember
- ○ rarely
- ○ never

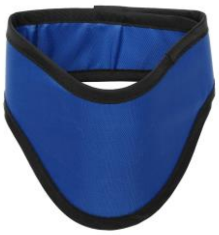

How often do you wear the overhead protective aprons? *هل ترتدي واقي الراس

- ○ always
- ○ most of the time
- ○ when I remember
- ○ rarely
- ○ never

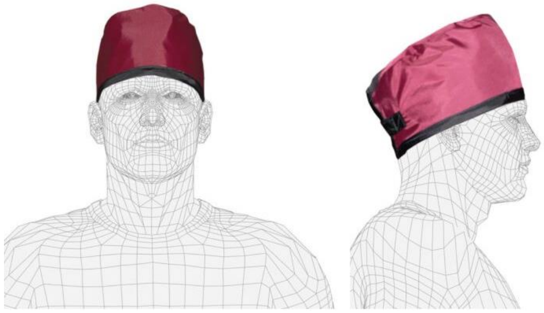

How often do you use the table's lower radiation shield (curtain) in the cath lab? هل تستخدم الواقي المثبت في القسطره*

- always
- most of the time
- when I remember
- rarely
- never

table lower curtain

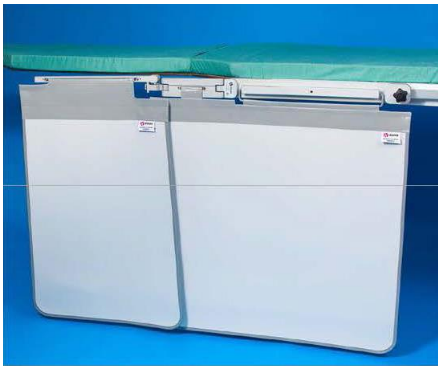

How often do you use the table's upper protection glass shield in the cath lab? هل تستخدم الزجاج الواقي*

- always
- most of the time
- when I remember
- rarely
- never

Table Glass shield

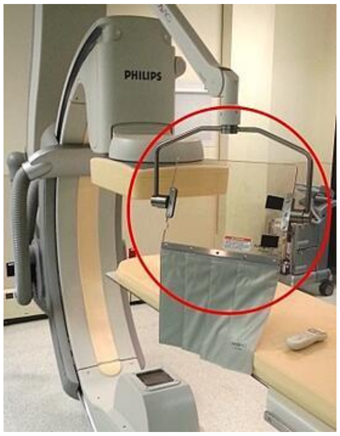

How often do you check your laboratory investigation to monitor the radiation effects? هل تتابع تحاليل الدم*

- always
- most of the time
- when I remember
- rarely
- never

did you have a health-related problem from radiation exposure (ex: leucopenia…)? هل تعرضت لمشاكل صحيه من قبل بسبب الاشعاع*

- ○ multiple times
- ○ once
- ○ never

if you answer yes, please mention health problem you had from radiation ما هي المشاكل الصحيه التي تعرضت لها بسبب الاشعاع ----- ان وجد

Did you have to refrain from working in the cath.lab before due to a radiation-related health problem? * هل امتنعت عن العمل بالقسطرة بسبب م شاكل الاشعاع من قبل

- ○ multiple times
- ○ once
- ○ never

if you answered yes for the previous question, mention the kind of health problem you had ما هي مشاكل الاشعاع التي تعرضت لها من قبل -- ان وجد

do you have a patient that has a radiation-induced health problem (ex: skin burn, ...etc) هل تعرف مرضي تعرضوا لمشاكل صحيه بسبب الاشعاع*

- ○ Yes
- ○ No

if you answer yes for the previous question, please mention what kind of incidence the patient had --- ----- ماهي مرضي المشاكل التي تعرض لها المرضي بسبب الاشعاع --ان وجد

Do you think you need a radiation awareness course (for those who do not have it before?) *هل تشعر انك بحاجه الي ورشه عمل للتوعيه بمخاطر الاشعاع

- ○ yes
- ○ No
